# Progress of Ship Exhaust Emissions in China’s Lijiang River: Current Status and Aftertreatment Technologies

**DOI:** 10.3390/toxics13050396

**Published:** 2025-05-15

**Authors:** Pengyu Liu, Bensen Xian, Mei Wang, Yong Xiao, Xiaobin Zhou, Dandan Xu, Yanan Zhang, Huili Liu, Shaoyuan Bai

**Affiliations:** 1 Guangxi Collaborative Innovation Center for Water Pollution Control and Water Safety in Karst Areas, Guilin University of Technology, Guilin 541006, China; liupengyu@mail.cgs.gov.cn (P.L.); xianbensen@glut.edu.cn (B.X.); liuhuili@glut.edu.cn (H.L.); baisy@glut.edu.cn (S.B.); 2 Institute of Karst Geology, Chinese Academy of Geological Sciences, Guilin 541004, China; 3 Hengsheng Water Environment Treatment Co., Ltd., Guilin 541100, China; wm905242022@163.com; 4 Guangxi Key Laboratory of Environmental Pollution Control Theory and Technology, Guilin University of Technology, Guilin 541006, China; a1003960187@gmail.com (Y.X.); zhouxiaobin@glut.edu.cn (X.Z.); zyanan@glut.edu.cn (Y.Z.)

**Keywords:** Lijiang River, inland shipping, exhaust emissions, air pollution control, environmental regulations, desulfurization and denitrification

## Abstract

Exhaust emissions from ships are significant threats to the environment and human health, necessitating effective control measures and treatment technologies. In response to the increasing stringency of emission regulations set by the International Maritime Organization (IMO) and national governments, the shipping industry must adopt advanced techniques to mitigate these emissions. The study focuses on the current status of exhaust pollution prevention and control on the Lijiang River and describes the latest progress in ship emission management. It summarizes the sources and hazards of nitrogen oxides (NO_X_), sulfur oxides (SO_X_), and particulate matter (PM) emitted from ships. The study introduces and compares several exhaust treatment key technologies for desulfurization, denitrification, and integrated desulfurization and denitrification to emphasize their principles, processes, and characteristics. It also demonstrates the future prospects for controlling exhaust gas pollution on inland ships and advocates for the development of integrated technologies that are efficient, space-saving, and cost-effective. The research aims to provide a valuable reference for inland ship exhaust pollution prevention and control.

## 1. Introduction

As a pillar of global trade, the shipping industry has an indispensable influence in promoting world economic growth. Nevertheless, the rapid development of inland river shipping has aroused great concern about its impact on air quality, because of its high maneuverability, wide voyage, and long duration of ship operation [1]. Inland ship benefits are based on environmental quality, scenic views of the nearby coastal areas, and the efficiency of the tourist services offered. They are, therefore, vulnerable to human activities that can adversely affect the ecosystem of the nearby areas. In addition, the poorer quality of marine fuels in comparison with vehicle fuels contributes to the increase in emissions. Currently, inland waterway vessels mainly use diesel engines as power sources, and the combustion of this oil releases a large number of harmful substances [2]. Annually, marine diesel engines emit around 20 million tons of nitrogen oxides (NO_X_), 10 million tons of sulfur oxides (SO_X_), and 1 million tons of particulate matter (PM) [3]. It is projected that by 2050, the international fleet’s global emissions of sulfur dioxide (SO_2_) and NO_X_ will amount to 25.9 and 38.8 million tons, respectively [4]. China’s inland waterway shipping consists mainly of small-tonnage and older vessels that have long relied on low-quality fuel oil, leading to more serious and complex environmental pollution problems. Emissions from ships can contribute significantly to air pollution, especially along inland waterways in densely populated cities. Air pollution is a pressing global issue that casts a shadow over public health and attracts more and more attention around the world [5]. According to statistical reports from the World Health Organization (WHO), accessible via its health statistics portal, the Global Health Observatory, air pollution is responsible for approximately 7 million deaths globally each year [6]. Alarmingly, WHO claimed that almost all the people in the world (99%) breathe more air than the limit, especially in low-income and middle-income countries in 2024. At the same time, the latest State of Global Air (SoGA) report in June 2024 indicated that air pollution contributed to roughly 8.1 million fatalities globally [7]. In order to tackle the issue, a Code for the prevention of air pollution from ships (MARPOL 73/78 Annex VI) has been put in place by the International Maritime Organization (IMO). Countries around the world have also issued regional standards for ship emissions. In recent years, the EU and the US updated the emission standards for inland waterway vessels, while China also issued the first national standard for the control of emissions from ship engines. In response to increasingly stringent emissions regulations, marine diesel engines utilize three main strategies for reducing emissions: fuel technology advancements, in-cylinder purification methods, and exhaust gas aftertreatment processes [8]. Emissions can be reduced by exhaust gas aftertreatment with little or no impact on engine power and fuel economy. Therefore, it is an urgent problem to solve ship emissions, especially the pollution control of SOx and NOx, which is of great significance for the construction of green harbors [9].

**Figure 1:**
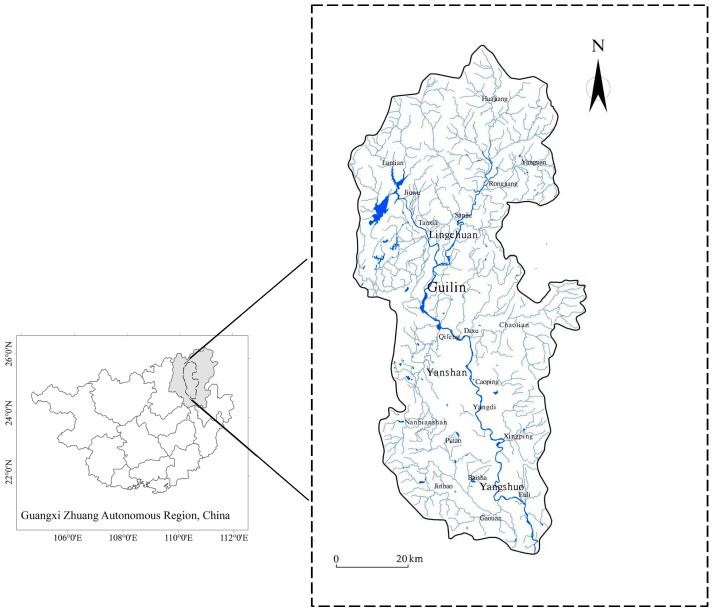
Map of Lijiang River Basin. (black lines represent watershed, grey blue lines represent tributary and blue lines represent main stream in the right figure).

The Lijiang River, a renowned tourist destination in China, lies within the city of Guilin, positioned in the northeastern part of the Guangxi Zhuang Autonomous Region. The watershed map of the Lijiang River is shown in Figure 1. The Lijiang River Basin serves as the central hub for Guilin’s socioeconomic expansion. This development has primarily centered on bolstering the agricultural and tourism sectors. In the Lijiang River Basin, tourism stands out as a pivotal industry. Tourism has boomed in the basin since 2000. In 2010, the tourist reception exceeded 18 million and the total tourism revenue was 13 billion, with a growth rate of 13.9% per annum. In 2023, an unprecedented 1.39 billion tourists visited the Lijiang River Basin, among whom the water tourist flow of the Lijiang River exceeded 11.1 million. Data statistics show Lijiang River tourist ships set sail a total of 83,000 times and sent 4.2 million tourists in 2023. Recent studies have reported a wide range of adverse impacts on ecosystems as well as on human well-being as a result of the development of tourism, including water pollution, air pollution, water scarcity, and cultural effects [10]. Shipping activities are recognized as an important factor contributing to global air contamination [11,12]. At present, more studies focus on ship exhaust emissions from ocean transportation and fewer reports on inland waterway vessel exhaust and regulation. Due to the limited range of activities and close to densely populated areas, the requirements for inland ship exhaust emissions are more stringent, especially for cruise ships operating in scenic spots. The gas pollution emitted by them is more harmful to human beings. Balancing the interplay between tourism growth and environmental preservation is a crucial matter to consider in the sustainable development of tourism [10].

Taking the Lijiang River as an example, this paper reviews the exhaust emission status of ships on the Lijiang River, the regulations on ship exhaust emissions, and the positive measures that have been taken to deal with ship exhaust contamination in the Lijiang River. In addition, this review also systematically summarizes the control methods for SO_X_ and NO_X_ emissions and the desulfurization and denitrification processes, as well as the applicability of these technologies in marine diesel engines. The primary aim of this study is to offer vital guidance for research on optimizing methods and decision-making systems for ship energy consumption and emissions, so as to help the green development of the shipping sector in the future.

## 2. Lijiang River Ship Emission Status and Regulation

### 2.1. Lijiang River Emission Status

Ship exhaust pollution in the Lijiang River mainly originates from ship moving, ship stopping, diesel cooking, and shipbuilding enterprises. As of September 2022, there are 255 passenger boats for tourism, 40 boats for government use, 33 ferries for transporting passengers, three bunker tankers for fuel supply, 88 vessels of other types, and 1300 rafts for tourism. There are four main passenger docks in the tourist area, including Binjiang Road Dock, Mopanshan Dock, Zhujiang Dock, and Yangshuo Longtoushan Dock. In addition, there are simple terminals in Daxu, Caoping, Yangdi, and Xingping that can dock small passenger ships. Currently, most of the ships sailing on the Lijiang River use traditional low-speed diesel engines, and there are fewer new energy vessels. Most ship engines use inexpensive and inferior fuels with heavy metals, which are much heavier and stickier, with longer carbon chains and higher sulfur content than the fuels used by land-based vehicles [13]. The exhaust of diesel engine ships not only includes carbon dioxide (CO_2_), which contributes to the global greenhouse effect, but also includes a lot of NO_X_, SO_X_, and PM [14,15]. NO_X_ and SO_X_ are major harmful emissions from marine diesel engines [16]. Lijiang waterway is the basis of sightseeing in tourist areas, and boat tour is the core tourism project. At present, there are more than a dozen visitor terminals in the Lijiang River. Only during the summer vacation in July–August 2024 did the Lijiang River cruise ships and rafts receive 1.97 million tourists. The huge number of tourists has caused a large amount of polluted gas emissions while contributing to the economic development of the Lijiang River [17]. In recent years, the daily average ship flow on the Lijiang River from Mopanshan Wharf to Longtoushan Wharf in Yangshuo is 70 times, while the daily average ship flow from Zhujiang Wharf to Yangshuo Watergate Wharf is 15 times. Each ship needs about 0.24 tons of diesel for a round trip, and 1 ton of diesel combustion produces about 3.2 tons of exhaust gas. Accordingly, tourist ships emit as much as 23,827.2 tons of exhaust gas into the atmosphere of the Lijiang navigation area in a year, which does not include indoor tourist ships [18]. The Lijiang River raft emits 372 t of CO_2_, 234 tons of carbon monoxide (CO), 10 tons of hydrocarbons (HC), 20 tons of NO_X_, 1.2 tons of sulfide, lead, solid particles, and other pollutants in a year. The annual emissions of SO_2_ and CO from cooking on cruise ships reach more than 9.2 tons [19]. Moreover, marinas along the Lijiang River are not equipped with perfect shore power facilities for the safe use of vessels during berthing, which contributes to the Lijiang River Basin’s exposure to the risk of environmental pollution from ship exhaust [20]. Furthermore, China’s Lijiang River ship exhaust treatment work started late, and the equipment was backward, so there was no perfect anti-pollution emergency response capacity of the ship [20]. The Lijiang River offers recreational amenities and scenic views to millions of residents and visitors. Information on the pollution from ship exhaust is also vital for policymaking in these heavily populated regions.

### 2.2. Lijiang River Emission Regulation

The shipping industry has been recognized as a major source of global environmental contamination. Thus, there is a need to regulate and enforce international standards for maritime discharges. The NO_X_ emission standards set by MARPOL apply to both existing and newly built marine diesel engines. The primary and secondary limits are applied to the whole world, but the tertiary standard applies only to NO_X_ emission control areas. Furthermore, it is important to limit the sulfur content of fuels, which significantly increases emissions of SO_X_ and respirable particulate matter (PM) [21].

To reduce the emission of pollutants by marine motors, China has developed as well as implemented several regulatory measures. For instance, China’s Stage I/II Standards, the IMO’s Annex VI Standards, and domestic Emission Control Areas. The Chinese Class I/II standards apply to both thrust and assist motors mounted on inland waterways and offshore vessels. Effective from 1 September 2018, every diesel motor fitted on Chinese-flagged vessels, as well as those imported vessels seeking to engage in internal commerce within the country’s ECAs, must comply with the Annex VI Tier II NO_X_ emission limit values [22]. From 1 March 2020, all vessels not equipped with exhaust gas purification systems will be prohibited from transporting fuel oil with a sulfur content of more than 0.5% [23]. The sulfur limit of 0.1% will also apply to ships entering inner channels and Hainan Island [24].

To improve the status of ship exhaust pollution in the Lijiang River, Guilin Maritime Safety Bureau has issued the “Guilin Maritime Safety Administration’s Three-Year Action Plan for Lijiang River Ship Pollution Prevention (2021.9–2024.8)”, and implemented some positive measures to ensure that the Lijiang River creates the “LiQing” brand [25].

#### 2.2.1. Promoting New Energy Ships

In the past, most of the Lijiang River docks used diesel generators, which were not only costly and inefficient but also produced a huge volume of waste gas and noise, becoming the source of air pollution in the Lijiang River. Guilin Power Supply Bureau actively publicizes and advocates the concept of replacing energy consumption with electricity. At the same time, they investigated the need for the renovation of the Lijiang River cruise terminal and conducted basic data analysis on the shore power projects at Zhujiang Terminal and Mopanshan Terminal. By designing shore power access solutions with different voltage levels, strong technical support is provided. In 2022, “Lijiang River Tourism Raft Inspection and Management Measures” were issued and represented the end of internal combustion engine rafts. Electric instead of fuel has become a major tool for the development of high-quality ship tourism in Guilin, Guangxi [20]. The optimization of ship power structures was promoted, and nearly 1200 rafts have completed the “diesel-to-electricity” conversion, and more than 1100 battery-powered rafts are put into daily operation, accounting for 93% of the total number of rafts currently in operation on the Lijiang River. The first voyage of the Lijiang River pure electric-powered sightseeing raft was held at the Yangdi Wharf on 20 April 2023 [26]. Guangxi’s new energy cruise ship “Guilin Tourism” started its first voyage on 16 May 2022 at Zhujiang Wharf. After the shore power transformation was implemented, the shore power consumption of 21 berths was 2520 Kw/h. Compared with diesel power generation, it can reduce energy costs by 2.9 × 10^5^ yuan per month, and carbon emissions can be reduced by 22.7 tons if calculated based on a total load of 6 kW for each ship at a berthing time of 10 h per day [27].

Meanwhile, the Guangxi Maritime Administration is working with several research institutes to actively promote liquefied natural gas (LNG) as ship power and to explore the possibility of using clean energy and new technologies such as electricity, hydrogen, natural gas, and methanol on Lijiang River vessels.

#### 2.2.2. Adjusting the Ship Structure

Ships that have exceeded their service life are required to be scrapped by law, and old and outdated ships will be eliminated [28]. The policy encourages the construction of energy-saving and environmentally friendly ships and the modification of pollutant storage and treatment equipment on ships. Ships that have been put into operation since 2021 must strictly implement related requests of the “Discharge Standard for Water Pollutants from Ships” (GB3552-2018) [29] and “Management Measures for the Prevention and Control of Water Pollution by Inland Vessels with a Gross Tonnage of less than 400 t”, and newly built substandard ships are strictly prohibited into the Lijiang River. To prevent pollution caused by ship exhaust on the Lijiang River, 199 substandard ships were regularized. At the same time, it promoted the phasing out and replacement of 13 obsolete tourist vessels under the ‘Xingping Fishing Village Tour’ project and actively promoted the adoption of shore-based electricity by docked vessels to reduce their reliance on marine fuels, thereby reducing the carbon footprint of the vessels. Old passenger ships over 20 years on the stream of the Lijiang River have been eliminated to upgrade the quality of tourist passenger ships and improve the ship safety and pollution prevention levels [30].

#### 2.2.3. Construction of Ship Exhaust Monitoring System

To make sure that ships comply with international regulatory standards, it is the importance of air pollution monitoring caused by ships by all law enforcement agencies. The introduction of an advanced vessel exhaust monitoring system makes the exhaust emission data visible and clear, which facilitates the management of law enforcement and environmental protection departments. The traditional monitoring methods (bench method and shipboard method) are to install fixed monitoring probes in places with dense ship traffic, such as docks, port areas, main bends, and river beaches. They are not cost-effective and lack sufficient competitiveness. Intelligent remote sensing monitoring equipment for ship exhaust can continuously measure and analyze the exhaust data from ships. Wireless radio frequency technology, ship exhaust telemetry [31], mobile measurements with unmanned aerial vehicles (UAVs) [32], UAV-based sensor sniffing method, remote monitoring [33], computer and network technology, which were used to strengthen the monitoring of ship exhaust, reduce ship exhaust emissions, and improve the comfort of coastal residents [34].

In 2021, the Guilin Maritime Safety Administration and technology manufacturers carried out a drone telemetry test of ship exhaust on the Lijiang River for the first time. The unmanned aerial vehicle remote sensing monitoring method is based on the gas monitoring equipment carried on board, using advanced infrared smoke sniffing technology to sample and analyze the smoke plumes emitted by ships. By measuring the values of sulfur dioxide, nitrogen oxides, and other pollutants in the exhaust gas, a preliminary analysis is conducted on whether the sulfur content, nitrogen oxides, and other indicators of ship fuel exceed the standard [35]. Technicians selected different ship types in the urban section and the waters of Zhujiang Wharf and carried out the remote measurement of the exhaust emissions of the ships under different driving conditions. Guilin Maritime Safety Administration and technology manufacturers supervise and manage the quality of ship fuel by means of drone sniffing and rapid detector sampling, urging ships to use compliant fuel, ensuring that sulfur oxides in ship exhaust can be reduced by more than 80% compared with the same period.

## 3. Ships’ Exhaust Emissions

**Figure 2:**
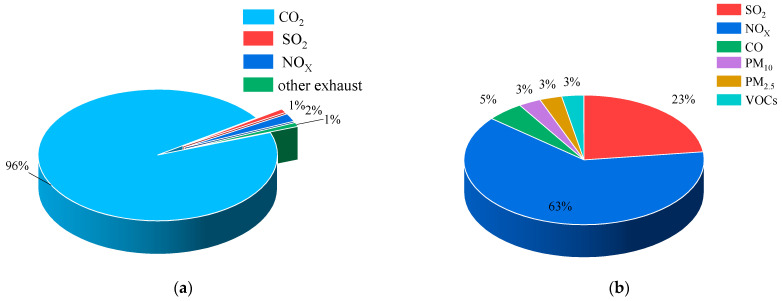
Ship exhaust emissions: Comparison of CO_2_, SO_X_, NO_X_, CO, and PM (**a**) containing CO_2,_ (**b**) containing CO_2_.

In China, vessel emissions are progressively emerging as a significant contributor to air pollution in advanced waterway transport areas such as the Pearl River Delta, the Yangtze River Delta, and the Bohai Rim region [36,37]. Exhaust emissions from ships mainly include SO_X_, NO_X_, PM, volatile organic compounds (VOCs), CO, CO_2_, HC, and ozone-depleting substances (ODS) [38,39]. Among these, SO_X_ and NO_X_ constitute the main components of ship emissions, which were listed as a priority for controlling ship exhaust pollutants by the IMO. Lu et al. studied the emission trends of SO_2_, NO_X_, PM_10,_ and other pollutants from 2000 to 2009, as well as the changes in their sources, using a dynamic approach in the Pearl River Delta. The results show that the marine mobile sources of SO_2_ emissions are mainly from the ship tailpipe, accounting for an average of 8%, with an average annual growth of 12%. Similarly, ship tailpipe emissions account for 69% of NO_X_ emissions in this region [40]. Fan et al. developed an automatic identification system (AIS)-based model for calculating the total emissions from ships in 2010 in the Yangtze River Delta region and in the sea area within 400 km of the East China Sea coastline. The SO_2_ and NO_X_ emitted from ships in key areas such as ports and shipping centers accounted for 15% and 14% of the anthropogenic emissions, far exceeding land-based emission levels [41]. Song et al. estimated the emissions of PM_10_, PM_2.5_, NO_X_, SO_X_, CO, HC, and CO_2_ from ships in the ports of Tianjin, Tangshan, Qinhuangdao, and Huanghua. The results showed that the emissions from ships increased with the distance of proximity to the port, and, in particular,, the concentration of emissions reached highest in the area within 15 nautical miles of the port. Based on the ship AIS data and fitting outcomes, the emissions from ships in the western part of Shenzhen port in 2018 were evaluated [42]. At the western part of Shenzhen port, the predominant ship exhaust component is CO_2_, which makes up 97% of total emissions. Followed by NO_X_ and SO_X_, they accounted for 2% and 1% of total emissions, respectively. Despite NO_X_ only accounting for 2%, its volume significantly surpasses that of other pollutants (Figure 2). By examining various ship types entering and exiting the western area of Shenzhen port, it is found that cargo ships have the highest contribution rate. Specifically, they account for 39.0% of CO_2_, 43.3% of SO_X_, 39.0% of NO_X_, 38.9% of CO, and 41.4% of PM emissions [43]. Among all ship types, the temporal emission intensity of CO_2_ from cargo ships sees the largest increase, followed by container ships and then tankers. When a cargo ship’s speed hits 10 knots, its CO_2_ emission rate is 13.18 tons per hour [44]. The temporal emissions of CO, CO_2_, NO_X_, PM_2.5_, and SO_2_ from cargo ships spike sharply when the speed reaches 15 knots, with container and tanker ships showing a similar pattern but to a lesser extent. Table 1 shows the contribution of air pollutant emissions from ships in major port cities of China measured over the years, where the pollutants with higher contributions are NO_X_, SO_X_, and PM.

### 3.1. Sources and Hazards of SO_X_

SO_2_ and SO_3_ are the major components of SO_X_, of which SO_2_ accounts for the vast majority (93%), and SO_2_ emissions are largely dependent on the sulfur content of the fuels used [47]. In a port site in Shanghai, SO_2_ accounted for 36.4% of ship exhaust emissions [48]. Sulfur is highly irritating and corrosive and is capable of damaging the human respiratory tract when its content exceeds the standard. It can also form sulfate aerosols within the atmosphere, which increases human health risks and the deterioration of land and waterborne environments [49]. Burning low-sulfur fuels is a straightforward approach to reducing sources of SO_X_ releases, but its price is higher than traditional heavy fuel oil, and the lower viscosity will lead to cylinder wear [50].

### 3.2. Sources and Hazards of NO_X_

NO_X_ is a very reactive gas that participates in a variety of chemical reactions in the atmosphere and is a greenhouse gas, mainly due to the combustion of nitrogen in the air when the engine is operated at high temperatures and high oxygen concentration [51,52,53]. The proportion of NO in NO_X_ emitted by the ship is 95%, and NO_2_ accounts for 5%. Moreover, the NO emitted from the engine is gradually oxidized to NO_2_ in the atmosphere, which adversely affects the breathing system, as well as reacting to form nitric acid and other compounds. Even though NO_2_ is approximately five times more soluble in water than NO under normal atmospheric conditions, typically only a portion of it can be removed [54]. Atmospheric NO_X_ emissions combine with chemical substances to form ground-level ozone as a result of heat and solar radiation. The shipping industry contributes significantly toward total NO_X_ releases, resulting in high ambient air concentrations of NO_X_ near port neighborhoods [55]. Overcoming this issue can be distilled into two main strategies: either further oxidizing NO_2_ into compounds with even greater solubility or employing a reducing agent to eliminate NO_2_ [56].

### 3.3. Sources and Hazards of PM

The high sulfur levels in the fuel result in high levels of PM and SO_X_ emissions from ship motors [57]. PM, which includes PM_2.5_ and PM10, primarily means smoke and dust particles from ship emissions, and may also result from the conversion of sulfur and NO_X_ [58]. PM emitted by ships has significant negative impacts on human health and the global climate [59]. Particulate matter emissions are associated with cardiac and lung diseases and, recently, with Alzheimer’s disease [53]. Particles may contain organisms associated with oncogenic and mutagenic activity, such as polycyclic aromatic hydrocarbons (PAHs) [60]. Ship PM_2.5_ emissions in the vicinity of port neighborhoods create racial and health risk disparities because low-income families are disproportionately represented in the affected population near the ports [61].

## 4. Ship Flue Gas Terminal Treatment Technology

As the IMO has clear limits on SO_X_ and NO_X_ emissions from marine diesel engines, desulfurization, and denitrification (FGD and N) technology has become the key to reducing atmospheric pollution from ship exhaust. Countries and regions around the globe are currently working on researching and improving flue gas FGD and N technologies to enhance ship exhaust treatment efficiency. Based on the chemicals utilized in the desulfurization process and the form of the end products, conventional desulfurization techniques can be categorized into wet, dry, and semi-dry methods. Numerous flue gas denitrification techniques have been devised, such as SCR, non-SCR, SNCR, catalytic oxidation, electron beam method, adsorption method, and microbial method [62].

### 4.1. Desulfurization Technology of Exhaust Gases

#### 4.1.1. Dry Exhaust Gas Desulfurization Technology

Dry exhaust gas desulfurization technology (FGD) implies that the entire reaction process is predominantly conducted in a dry condition [63]. The principle of dry desulfurization is that CaO or Ca(OH)_2_ reacts with sulfide to form CaSO_3_ and CaSO_4_ to achieve desulfurization. Compared with wet flue gas desulfurization, this method requires lower investment and operational expenses, and dry desulfurization products are more readily reprocessed [64]. Osaka et al. studied the capture of SO_2_ by a dry desulfurization filter of manganese oxide. The technology with this material has a large enough SO_2_ adsorption rate to capture significant amounts of SO_2_ gas [65]. However, the application of dry FGD on ships is affected by the limited space available and the difficult treatment of by-products generated by the reaction, so it is less suitable for the desulfurization of ship exhaust. Due to the lower response rate and higher residence time, the size of a dry FGD plant is about twice that of a wet FGD.

**Figure 3:**
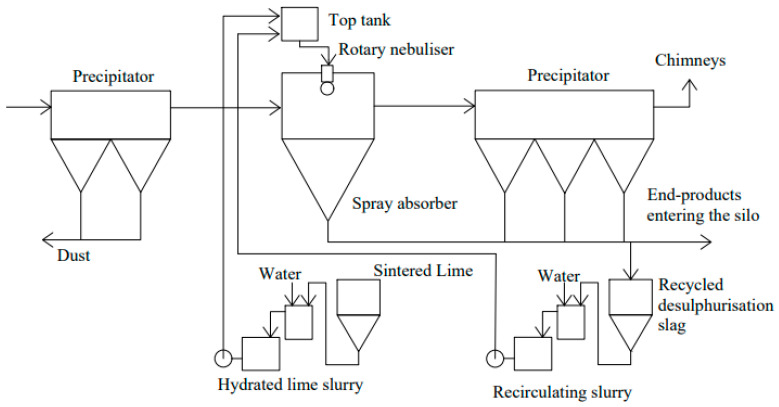
The main process of rotary spray drying technology.

Spray Dryer Absorber (SDA) is one of the dry desulfurization technologies, which uses Ca(OH)_2_ to neutralize sulfur oxides [66]. In this process, exhaust gases from ships interact with an atomized limestone slurry in a rotating spray drying tower to achieve effective neutralization of SO_2_ (Figure 3). As the solvent evaporates, the reaction produces a dry solid by-product (CaSO_3_) [67]. The SDA system is used in conjunction with a bag filter to achieve simultaneous desulfurization and dedusting, resulting in an integrated flue gas purification effect. In addition, SDA technology eliminates facilities such as post-treatment systems, post-treatment facilities, and flue gas heating links. The treatment process is suitable for ships with sufficient space and low requirements for by-product treatment, such as large cargo ships or tankers. It utilizes Ca(OH)_2_ to react with SO_2_ to achieve 90% to 95% desulfurization efficiency, with both dust removal effect, low energy consumption, and simplified equipment. However, due to the large size of the equipment, low reaction rate, and complex by-product treatment, it is not suitable for small and medium-sized ships with space constraints [68]. The reaction mechanisms for the reduction of SO_2_ can be summarized as follows [69]:

SO_2_ + Ca(OH)_2_→CaSO_3_ + H_2_O (1)

Some of the SO_2_ completes the following reaction:

SO_2_ + 1/2O_2_ + Ca(OH)_2_→CaSO_4_ + H_2_O (2)

#### 4.1.2. Wet Exhaust Gas Desulfurization Technology

Wet flue gas desulfurization technology is commonly performed in many thermal power plants and has a high desulfurization efficiency. Alkaline solutions (Ca(OH)_2_, Mg(OH)_2_) are used as neutralizing agents to absorb sulfur oxides that come into contact with the exhaust gas emitted from ships, thus scrubbing them to generate sulfite slurry. The desulfurization efficiency of wet exhaust gas desulphurization technology is usually over 95%. Wet FGDs mainly include open seawater scrubbing FGDs, closed cycle scrubbing FGDs (magnesium, sodic alkali), mixed scrubbing system FGDs, lime gypsum, and ammonia [70].

(1) Seawater exhaust gas desulfurization

**Figure 4:**
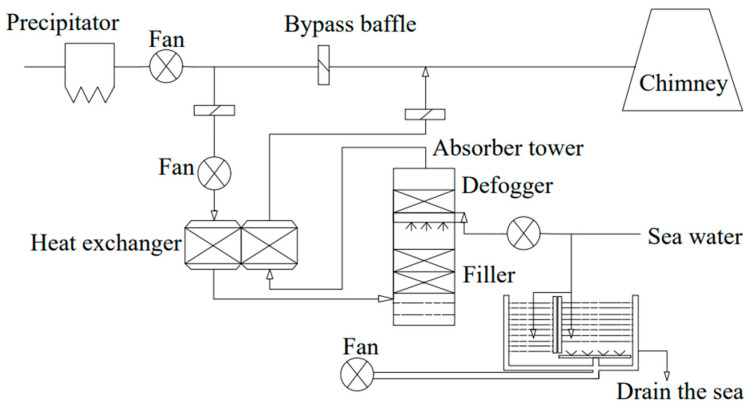
Process flow chart of seawater desulfurization.

The open seawater scrubbing desulfurization system is relatively straightforward. Seawater desulfurization uses seawater as a scrubber, where the naturally occurring basic components(Na_2_CO_3_, NaHCO_3_) combine with SO_X_ from ship emissions to form bisulfates and sulfates, which are discharged directly to the sea. Seawater desulfurization is suitable for ocean voyages. It essentially discharges SO_2_ emitted into the atmosphere into seawater, and the main process is shown in Figure 4. The seawater desulfurization process solely requires seawater and air, with no additional chemicals needed, and generates no solid or liquid waste. However, this process has drawbacks such as a relatively low overall sulfur dioxide removal efficiency and the necessity for a large volume of seawater when treating gases with high sulfur content [71,72,73]. Zhao et al. [74] discussed the feasibility of using seawater resources combined with BAD (C_12_H_25_NO) for simultaneous desulfurization and denitrification of ship exhaust. The average removal rate of SO_2_ and NO_X_ was 97.10% and 74.28%. Because of the low alkalinity of natural seawater with limited buffering ability between acid and alkali, this is only applicable to ship exhausts containing low-sulfur content. According to “2020 Implementation Plan of International Marine Fuel Oil Sulphur Limitations”, it is prohibited for ships to discharge scrubber water treated by an open exhaust gas cleaning system in the air pollutant emission control zones delineated in China’s maritime areas [75]. Therefore, seawater scrubbing desulfurization is no longer suitable for ship tail gas treatment under the new standard. The SO_2_ removal mechanism is as follows [76]:

SO_2_ + H_2_O→H_2_SO_3_ (3)

H_2_SO_3_↔HSO_3_^−^ + H^+^ (4)

HSO_3_^−^ + H_2_O↔SO_3_^2−^ + H_3_O^+^ (5)

HCO_3_^−^ + H^+^↔CO_2_ + H_2_O (6)

H_2_CO_3_ + H_2_O↔HCO_3_^−^ + H_3_O^+^ (7)

HCO_3_^−^ + H_2_O↔CO_3_^2−^ + H_3_O^+^ (8)

CO_3_^2−^ + 2H^+^↔CO_2_ + H_2_O (9)

**Figure 5:**
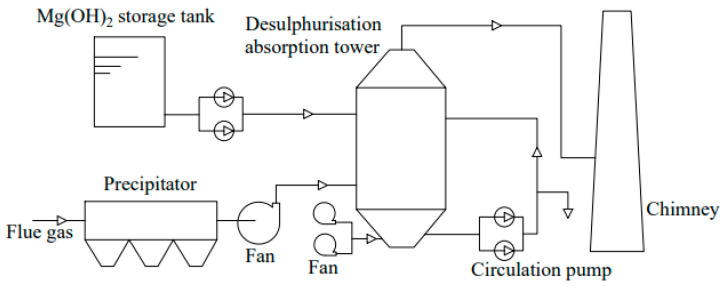
Process flow chart of magnesium oxide desulfurization.

The method uses magnesium oxide as the desulfurizing agent. As shown in Figure 5, a magnesium hydroxide (Mg(OH)_2_) solution is first prepared in the pulping equipment, followed by the reaction of Mg(OH)_2_ and SO_2_ in the absorption tower. Lastly, the absorption liquid and sediment are treated [77]. Regarding the byproducts of Mg-based desulfurization, commonly referred to as magnesium gypsum, magnesium sulfate (MgSO_4_), and magnesium sulfite (MgSO_3_) constitute the primary components [78,79]. The magnesium sulfate is dehydrated and dried and can be used for other purposes. China ranks among the top magnesite-producing countries globally [80]. The solid magnesium oxide is small, safe, cheap, environmentally friendly, and has a large adsorption capacity. The magnesium oxide wet flue gas desulfurization technology represents an economical and effective method for desulfurization. It is characterized by high desulfurization efficiency, low investment costs, compact unit size, and minimal environmental impact. Magnesium oxide, nevertheless, is more expensive and has a concentrated origin. Various manganese oxide samples with different physical and chemical structures were prepared using template, precipitation, and microwave methods for the desulfurization of ship exhaust. Manganese oxide prepared using the template method presented a rich 3D pore structure, a large specific surface area, and excellent desulfurization performance [81].

(2) Sodium alkali exhaust gas desulfurization

Sodium alkali desulfurization is a wet FGD process, and the main chemical equations are [82]:

2NaOH + SO_2_→Na_2_SO_3_ + H_2_O (10)

Na_2_SO_3_ + H_2_O + SO_2_→NaHSO_3_ (11)

Na_2_SO_3_ + SO_2_→Na_2_S_2_O_5_ (12)

(Since there is some oxygen in the flue gas)

2Na_2_SO_3_ + O_2_→2Na_2_SO_4_ (13)

**Figure 6:**
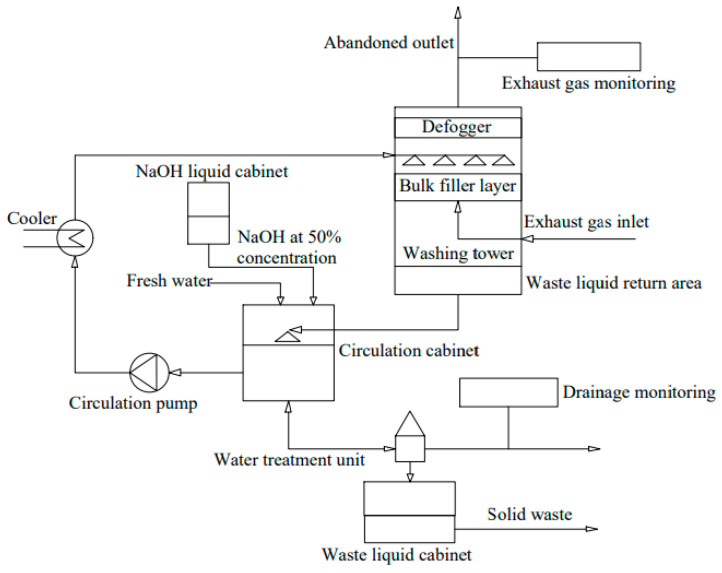
Schematic diagram of the sodium alkali desulfurization process.

In the reaction process, NaOH is used as the initial absorber, and it reacts with SO_2_ in ship exhaust gas to generate Na_2_SO_3_, NaHSO_3_, Na_2_S_2_O_5,_ and Na_2_SO_4_ to achieve the purpose of desulfurization, sequentially [83]. The temperature directly affects the removal efficiency of the desulfurizing agent [84]. NaHSO_3_ absorption solution after desulfurization is evaporated, filtered, and cooled to precipitate Na_2_SO_3_, which is soluble in the condensate, and then is pumped into the scrubber tower for recycling. Sodium alkali has a strong affinity with SO_2_ and can achieve a high desulfurization rate [85]. Desulfurization produces sodium salt, which can avoid clogging in the absorption tower. However, NaOH is hazardous and corrosive and should not be stored in large quantities on board. The treatment process is suitable for large ocean-going vessels and less suitable for small ships or inland waterway vessels. When the liquid–gas ratio of seawater sodium alkali absorption solution is 5 L/m^3^, the desulfurization rate can reach more than 99% [86]. It can efficiently remove SO_2_ from the exhaust gas, but its application is limited due to the limitations of high cost, high corrosiveness, and non-recyclability of the desulfurizer [87]. The process flow is depicted in Figure 6.

(3) Limestone–gypsum exhaust gas desulfurization

**Figure 7:**
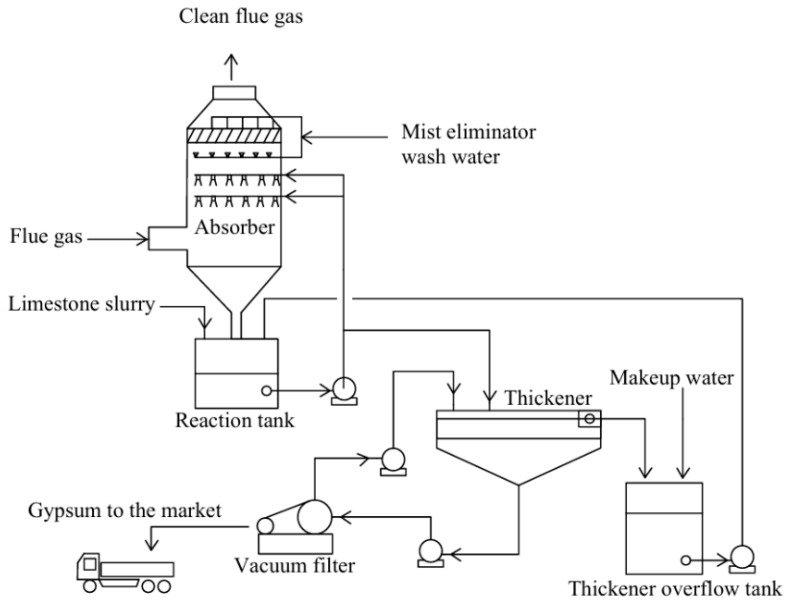
Typical process of limestone–gypsum desulfurization.

In the limestone–gypsum wet FGD process, SO_2_ in the emitted ship exhaust reacts with calcium hydroxide, Ca(OH)_2_, to produce calcium sulfite (CaSO_3_). Subsequently, the remaining CaSO_3_ is completely converted to calcium sulfate (CaSO_4_) after sufficient contact with oxygen in the air (Figure 7). The main chemical equation is [84]:

SO_2_ + Ca(OH)_2_→2CaSO_3_ + H_2_O (14)

CaSO_3_ + 2H_2_O→CaSO_4_·2H_2_O (15)

2CaSO_3_·1/2H_2_O + O_2_ + 4H_2_O→2CaSO_4_·2H_2_O + H_2_O (16)

The core part of the reaction process is that the flue gas enters the interior of the absorber tower, and the desulfurization reaction occurs by mixing the desulfurizing agent with the flue gas through a spray system to remove SO_2_ and SO_3_ [88]. The limestone–gypsum process allows the oxidation of CaSO_3_ to be controlled, thus ameliorating the problem of fouling and clogging of the absorber tower and pipework in the conventional lime process. Limestone–gypsum wet FGD technology is suitable for large ships, as it can efficiently remove SO_2_ from the exhaust gas and convert it into gypsum with high desulfurization efficiency (more than 90%). However, the large size of the equipment, its susceptibility to wear and clogging, and complicated wastewater treatment limit its wide application on ships [89].

(4) Ammonia exhaust gas desulfurization

**Figure 8:**
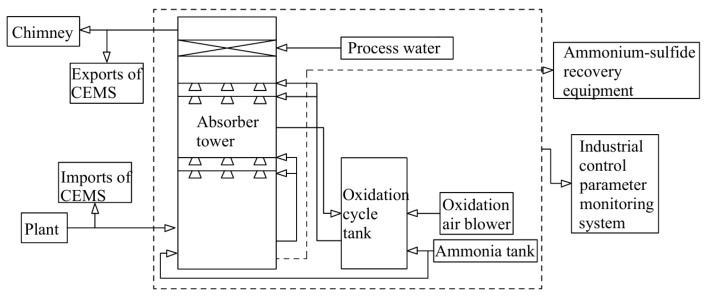
Process flow chart of ammonia desulfurization system.

The ammonia FGD technology, which has intellectual property rights in China, can achieve a 98% desulfurization rate and a 50% denitrification rate under optimal operating conditions [90]. Ammoniated desulfurization can ultimately convert SO_2_ from exhaust gases into an environmentally friendly ammonium sulfate ((NH_4_)_2_SO_4_) fertilizer product. The flue gas is sprayed and scrubbed into an ammonia suction tower for desulfurization of the mother liquor, followed by acid digestion and neutralization of the mother liquor to produce (NH_4_)_2_SO_4_, and the gas is discharged after meeting the standard [91]. Ammonia exhibits high reactivity, a rapid reaction rate, a straightforward process, is resistant to fouling and clogging, and is easy to start, stop, and maintain [92]. As the production of synthetic ammonia gas increases, ammonia flue gas desulfurization technology has seen rapid development and garnered extensive attention in China. Ammonia can be used by low- and medium-speed engines to reduce emissions by 90% [93]. The process flow is illustrated in Figure 8. The technological principle is outlined as follows [94]:

2NH_3_ + H_2_O + SO_2_→(NH_4_)_2_SO_3_ (17)

(NH_4_)_2_SO_3_ + SO_2_→2NH_4_HSO_3_ (18)

NH_4_HSO_3_ + NH_3_→(NH_4_)_2_SO_3_ (19)

2(NH_4_)_2_SO_3_ + O_2_→2(NH_4_)_2_SO_4_ (20)

### 4.2. Denitrification Technology of Exhaust Gas

#### 4.2.1. Selective Catalytic Reduction (SCR) Exhaust Denitrification Technology

**Figure 9:**
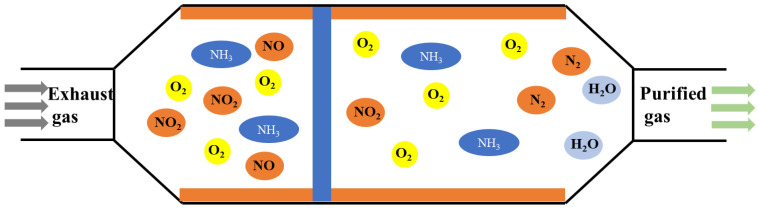
Schematic diagram of SCR catalytic mechanism [105].

SCR technology is considered to be one of the most advanced and effective ways to reduce NO_x_ emissions by using reductants in the presence of oxygen. It is mainly used in ships with four-stroke engines [95]. SCR technology employs carbamide or ammonia solution (NH_3_-SCR) as a reducing agent to transform NO_X_ into N_2_ and H_2_O, as shown in Figure 9. For SCR reactions, an aqueous ammonia solution is the preferred choice. In addition to NH_3_-SCR, carbon monoxide (CO-SCR), hydrogen (H_2_-SCR), and hydrocarbons (HC-SCR) are also used as marine SCR technology suitable reduction agents [96]. Selecting the right catalyst is crucial for the operation and effectiveness of an SCR system. Various types of catalysts are employed in NH_3_-SCR processes, including precious metals, metal oxides such as titanium, vanadium, iron, activated carbon (which can be pulverized high carbon coal or coke mixed with inert elements), and zeolites (which are crystalline, highly porous, and naturally or synthetic aluminosilicates) [97]. Vanadium pentoxide (V_2_O_5_) is the most frequently used industrial catalyst for the elimination of NO_x_ and has been commercialized with titanium dioxide (TiO_2_) as a carrier, owing to its excellent sulfur resistance and long-term stability [98]. SCR catalysts for marine operations are often V_2_O_5_ catalysts that exhibit high activity and are sulfur resistance [99,100,101]. The reaction equation with ammonia as a reducing agent is as follows:

4NH_3_ + 4NO + O_2_→4N_2_ + 6H_2_O (21)

8NH_3_ + 6NO_2_→7N_2_ + 12H_2_O (22)

**Figure 10:**
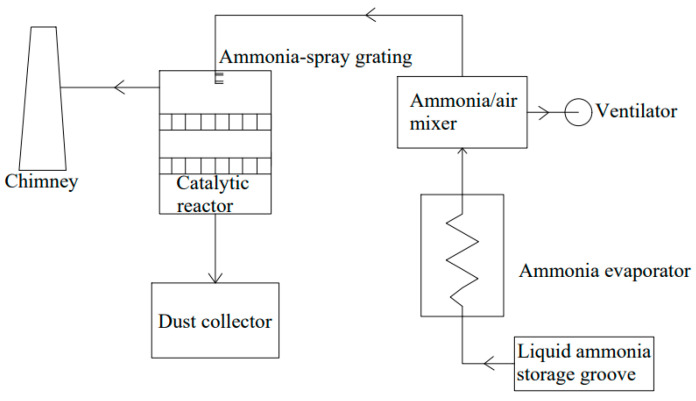
Process flow chart of selective catalytic reduction.

SCR technology can be compatible even with remnant marine fuels, as the integral stationary base has square holes, and the holes are large enough to prevent plugging and toxicity [102]. SCR technology is favored by the market because of its no by-products, no secondary pollution, simple equipment design, mature technology, high denitrification efficiency, stable operation, and easy maintenance. But SCR catalyst materials are easy to scale, block, and poison. The method is mainly applicable to ocean-going vessel navigation, but it is seldom used by two-stroke ships sailing in the Lijiang River inland waterways. Significant NO_X_ reductions of nearly 90% are reported for ships using SCR [103,104]. However, on the two ferry motors with SCR units, the conversion of NO_X_ ranged from 36% to 94%, according to the temperature of the catalyst [83]. SCR system’s optimal operating temperature is 300–400 °C; if the system temperature is too high or too low, it cannot function properly. The process flow is shown in Figure 10.

#### 4.2.2. Selective Non-Catalytic Reduction Denitrification Technology

Selective non-catalytic reduction (SNCR) involves reducing NO_X_ to N_2_ within a temperature range of 800–1100 °C by reacting with an amine reagent in the presence of oxygen. Since this process occurs at elevated temperatures, it does not need a catalyst to trigger the reaction. The reagent, either ammonia or urea, can be directly injected into the combustion chamber. Urea-based SNCR offers advantages over ammonia-based systems because urea is safer to store and handle due to its non-toxic nature and lower volatility. However, urea is more expensive than ammonia, and the urea reduction process generates more nitrous oxide compared to the ammonia reduction process [106]. The main reaction mechanism is listed as follows:

(23) $$4{\mathrm{NH}}_{3}+6\mathrm{NO}\to 5N_{2}+6H_{2}$$

CO(NH_2_)_2_ + 2NO + 0.5O_2_→2N_2_ + CO_2_ + 2H_2_O (24)

**Figure 11:**
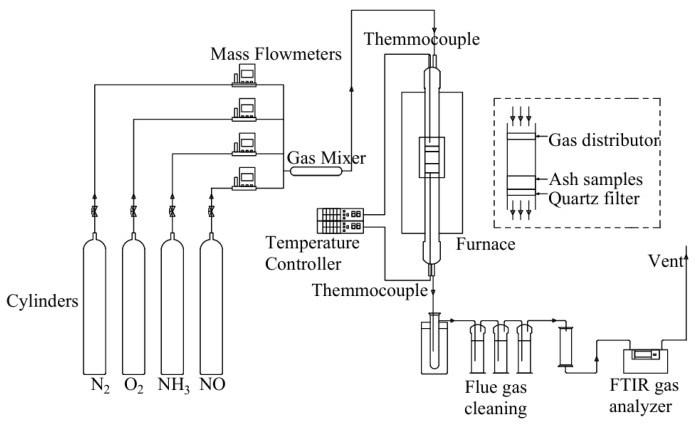
Process flow chart of selective non-catalytic reduction.

Technology has gained traction because of its relative simpleness, lack of need for a catalyst system, easy arrangement in existing power stations, wide application range, low cost, immunity to fly ash, and can be used in conjunction with other NO_x_ emission control technologies [107]. In commercial applications, the NO_x_ concentration in the gas emitted at the stack is 60% lower with SCR than with SNCR, suggesting that SNCR uses significantly more reductant than SCR, typically three to four times more than SCR [108]. Since the emission temperature of ship exhaust is 300–400 °C, and the temperature window of SNCR denitrification is generally 800~1100 °C, the method is not applicable to the treatment of ship exhaust. The SNCR denitrification process is shown in Figure 11.

#### 4.2.3. Ozone Oxidation Denitrification Technology

Based on the highly efficient oxidizing capacity of O_3_, ozone oxidative denitrification technology converts nearly 95% of the nitric oxide (NO) in ship exhaust to higher order nitrogen oxides (NO_X_) that are soluble in water and react with alkaline solutions, followed by subsequent treatment using solution absorption [109]. The method boasts strong selectivity and rapid oxidation rates [110]. Additionally, it is capable of oxidizing mercury and volatile organic compounds in flue gas, thereby achieving integrated denitrification [111,112,113]. The specific chemical equation is:

O_3_ + NO→NO_2_ + O_2_ (25)

O_2_ + 2NO_2_→2NO_3_ (26)

NO_2_ + NO_3_→N_2_O_5_ (27)

N_2_O_5_ + 2OH^−^→2NO^3−^ + H_2_O (28)

**Figure 12:**
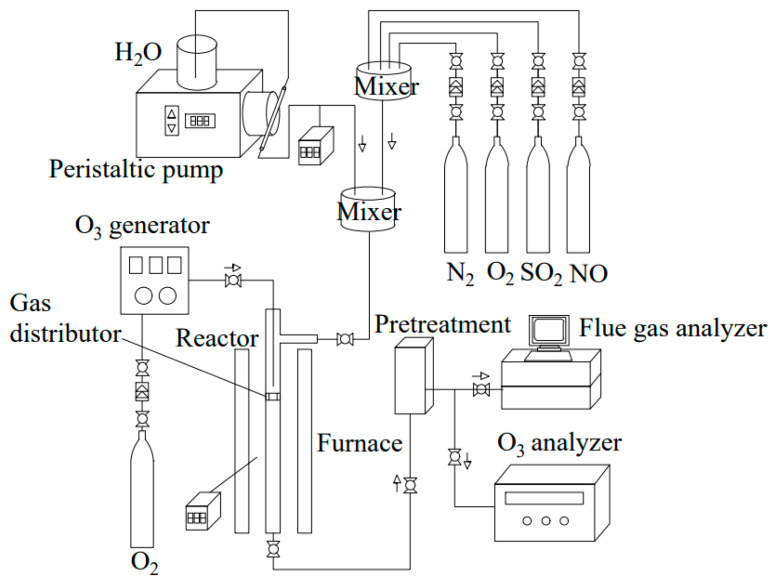
Process flow chart of ozone denitrification [117].

Wang et al. [114] investigated the oxidation of NO by O_3_ using simulated fuel gas. Their findings indicated that the oxidation efficiency exceeded 90% at a reaction temperature of 150 °C. Xia et al. [115] studied co-oxidized O_3_ with NaClO scrubbing liquid for ship exhaust, using the liquid to lower the temperature of the exhaust gas, less decomposition of O_3,_ and optimize the reaction effect, while reducing the consumption of electrical energy. The ozone denitrification equipment is divided into three main segments, which are the ozone preparation system, ozone oxidation system, and absorption system (Figure 12). Both oxidation and adsorption processes have a direct impact on the efficiency of NO_X_ removal and the operational economy [116]. Currently, O_3_ is typically produced through dielectric barrier discharge. But only a small fraction of O_2_ molecules is ionized to form O_3_, leading to high energy consumption, significant O_2_ usage, and high costs [117,118,119]. These factors limit the popularization and application of O_3_ oxidation denitrification technology.

### 4.3. Integrated Exhaust Gas Desulfurization and Denitrification Technology

#### 4.3.1. Electrostatic Spray Technology

**Figure 13:**
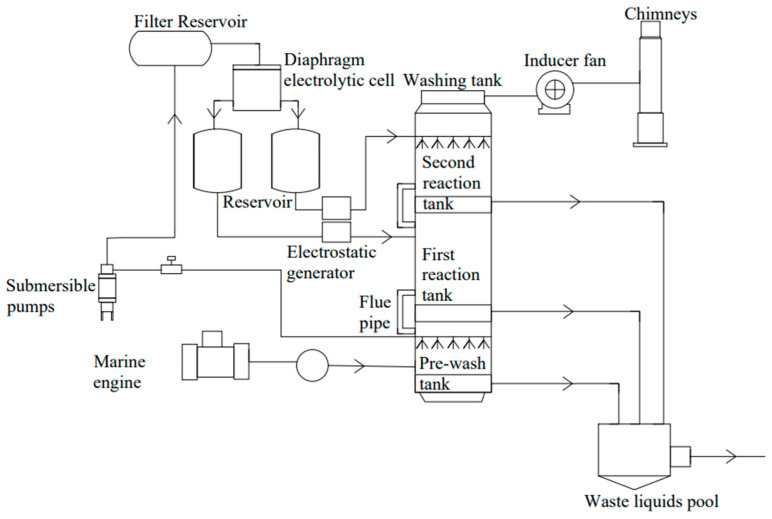
Process flow chart of desulfurization and denitrification integration with electrostatic spray technology.

Electrostatic spraying is used to remove pollutants from ship exhaust gas, based on traditional wet scrubbers. The dust is charged by corona charging and the spray droplets are directly charged by an electrostatic generator (Figure 13). The opposite charges between the two generate electrostatic attraction, so the droplets can more easily combine with pollutants, thereby improving the efficiency of the droplets in removing fine dust [120]. Oh et al. assessed the applicability of electrostatic spray technology for eliminating particulate matter produced following the electron beam treatment of NO_X_ and SO_X_. In conditions with low particle number concentration (additive: NaOH, gas: NO, and NO_2_), even at an applied voltage of 5 kV, high removal efficiencies (ranging from 86.1% to 96.5%) were achieved [121]. The electrostatic atomization equipment is added to the traditional water washing tower. The device has two characteristics: (1) It uses seawater for desulfurization and denitrification, and the raw materials are inexhaustible. At the same time, it reduces the space occupied by traditional desulfurization and denitrification reactants, saves costs, and improves efficiency. (2) The introduction of electrostatic atomization equipment into ship exhaust treatment can better remove dust and make the droplets uniform and fine, increase the spatial density of the droplets, thereby increasing the contact reaction surface with sulfides and nitrates, and improve the capacity and efficiency of traditional desulfurization and denitrification equipment [122].

#### 4.3.2. Plasma Oxidation Process

**Figure 14:**
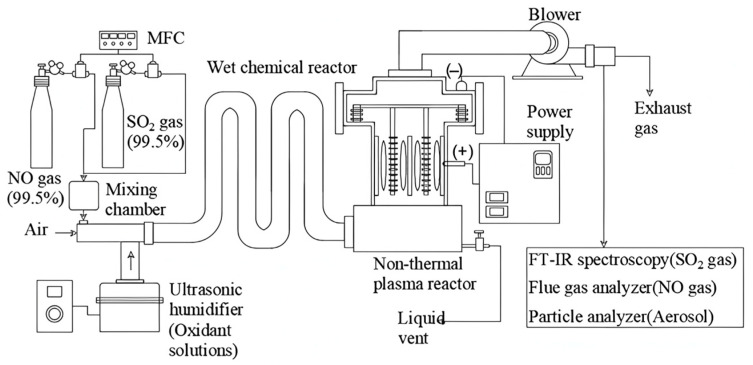
Facility diagram of wet scrubber combined with plasma electrostatic precipitator [126].

Plasma oxidation mainly employs high-energy electrons to energize, ionize, or even break apart SO_2_ and NO_X_ molecules in flue gas, thereby producing a substantial amount of reactive particles, such as ions and radicals. The plasma oxidation process includes electron beam irradiation and a pulsed corona-induced plasma chemical process, which removes NO_X_ and SO_2_ by oxidizing NO_x_ and SO_2_ to highly valent oxides. (NH_4_)_2_SO_4,_ and ammonium nitrate (NH_4_NO_3_) fertilizer are formed when ammonia is injected (Figure 14) [123,124]. However, the method has also technical challenges: (1) the NO_x_ removal efficiency may drop to 0 or a negative value under certain oxygen concentrations; (2) CO_2_ and water vapor in the tail gas can also have a negative impact on the effectiveness of desulfurization and denitrification. Therefore, for the successful application of plasma oxidation for desulfurization and denitrification of ships, its use in combination with adsorption and catalysis needs to be continuously explored [111]. Cui et al. devised an integrated system for the removal of NO and SO_2_, combining a wet electrostatic precipitator (WESP) with dielectric barrier discharge (DBD) [125]. The oxidizing agents produced in the DBD process oxidize SO_2_ and NO. In the presence of water vapor in the flue gas, these oxidation products can further transform into acid mist, which the WESP can subsequently absorb and capture, along with any residual free radicals. The system demonstrated enhanced removal capabilities, achieving a peak removal efficiency of 98.9% for SO_2_ and 87.1% for NO. The integration of plasma technology significantly boosts the removal efficiency of SO_2_ and NO_X_. However, as evident from Figure 14, the addition of the electrostatic component complicates the system and leads to increased investment and operational costs.

#### 4.3.3. Oxidation–Reduction–Absorption Technology

The SO_3_^2−^ based oxidation–reduction–absorption process is preferred for its high removal efficiency and the ease with which it can modify existing wet FGD and N equipment [122]. Approximately 90% of the NO_X_ in flue gas is NO, which has limited solubility in solutions [127]. Therefore, NO is typically oxidized using oxidizing agents in the initial stage of the oxidation–reduction–absorption process [128]. Subsequently, the higher-valent NO_X_ can be more readily removed by adsorbents like Na_2_S, NaOH, or (NH_4_)_2_SO_3_ [129,130,131]. Reports indicate that Na_2_SO_3_ solutions demonstrate outstanding efficiency in removing NO_X_ and SO_X_ [132,133]. However, certain issues warrant attention: firstly, sulfite is prone to oxidation by O_2_ in the presence of NO_X_ as a catalyst, which results in reduced effectiveness for NO_X_ absorption. Secondly, there is competitive absorption between NO_X_and SO_2_ when using Na_2_SO_3_. SO_2_ can also consume a significant amount of SO_3_^2−^, leading to a considerable decrease in the amount of NO_x_ absorbed [134]. NaClO_2_ and Na_2_S_2_O_3_ were used for oxidizing and reducing sections to remove SO_2_ and NO_X_ from simulated ship emissions. It was shown that although the more well-known sulfite (Na_2_SO_3_) was about 15% more efficient in removing NO_2_ compared to thiosulfate, the latter had a very low reactant consumption rate [135]. The reaction equation is as follows:

2NO + O_2_→2NO_2_ (29)

NO_2_ + 2NaOH→NaNO_2_ + NaNO_3_ + H_2_O (30)

SO_2_ + H_2_O→HSO_3_^−^ + H^+^ (31)

HSO_3_^−^→SO_3_^2−^ + H^+^ (32)

#### 4.3.4. UV/Chlorine Advanced Oxidation Technology

**Figure 15:**
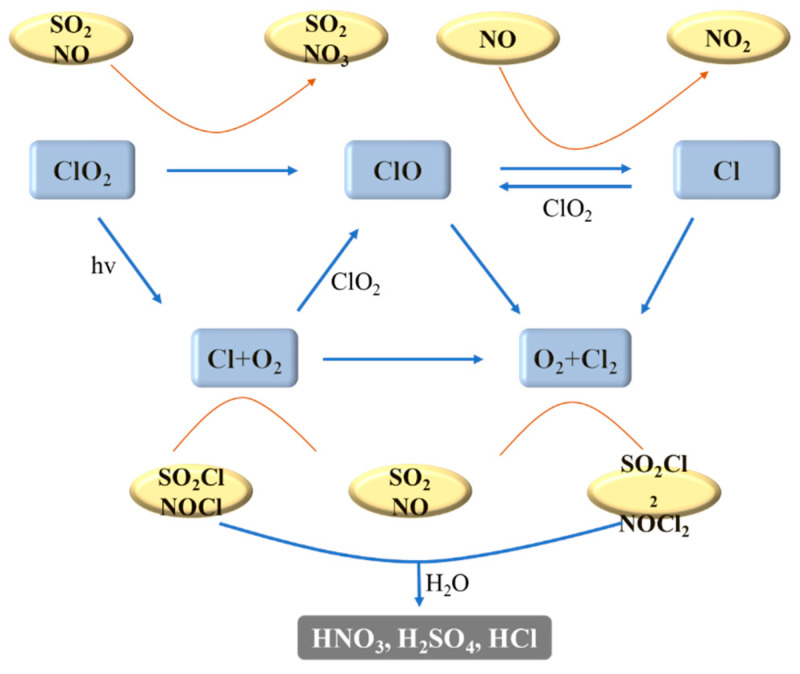
Removal process of SO_2_ and NO_X_ by Cl_2_ and ClO_2_ under UV [139].

In the UV/chlorine treatment involving active chlorine solution, the effective components of active chlorine, HOCl and OCl^−^, generate several free radicals via photolysis reactions, including hydroxyl (·OH) and chlorine (Cl·) radicals (Figure 15). Due to the strong and non-selective oxidation properties of these free radicals, it has been reported that UV/chlorine can oxidize NO from flue gas through a wet scrubbing process. The denitrification efficiency of effective chlorine in electrolyzed seawater is extremely low in the absence of light [136]. A significant synergistic effect on NO absorption was attained by integrating UV with electrolyzed seawater. At a pH of 6, NO was primarily removed by the HOCl present in the electrolyzed seawater.

NO(g)↔NO(aq)(33)

NO(aq) + HOCl↔NO_2_ + H + Cl^−^ (34)

3NO_2_(aq) + H_2_O↔2H^+^ + 2NO_3_^−^ + NO(aq) (35)

2NO_2_(aq) + H_2_O↔HNO_2_ + H^+^ + NO_3_^−^ (36)

HNO_2_↔H^+^ + NO_2_^−^ (37)

NO_2_^−^ + HOCl→NO_3_^−^ + H + Cl^−^ (38)

Even though SO_2_ competes with NO for oxidants, the concentrations of UV-induced oxidants in electrolyzed seawater were ample to absorb both NO and SO_2_ concurrently in the photochemical bubble reactor. As the SO_2_ concentration rose from 200 to 1000 ppm, the NO absorption rate saw a slight increase from 2.9 × 10^−5^ to 3.0 × 10^−5^ mol/(m^2^·s) [137].

SO_2_(g)↔SO_2_(aq) (39)

SO_2_(aq) + H_2_O↔HSO_3_^−^ + H^+^ (40)

HSO_3_^−^ + HO·↔SO_4_^2−^ + 2H^+^ (41)

HSO_3_^−^ + HOCl↔SO_4_^2−^ + 2H^+^ + Cl^−^ (42)

The removal rate of SO_2_ from the ship’s exhaust gas by the effective chlorine solution can always be 100% when the pH is between 3 and 10. When the pH of the effective chlorine solution was maintained between 4 and 7, it was able to generate free radicals with high activity, which significantly enhanced the denitrification effect [131]. As the power of the UV irradiation UV lamp set increased, the concentration of active free radicals generated by the photolysis of effective chlorine increased, the oxidative property of the solution was enhanced, and the removal rates of NO and NO_X_ increased close to linearly [138]. When practically applied to the purification of ship exhaust, the denitrification efficiency can be improved by implementing a multi-stage treatment strategy.

#### 4.3.5. Photocatalysis

**Figure 16:**
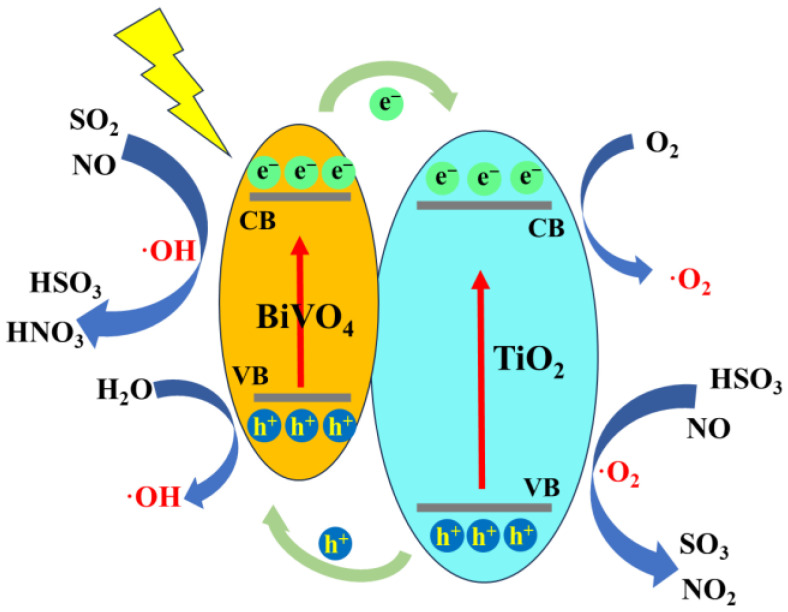
Proposed mechanism of photocatalytic reaction [142].

**Figure 17:**
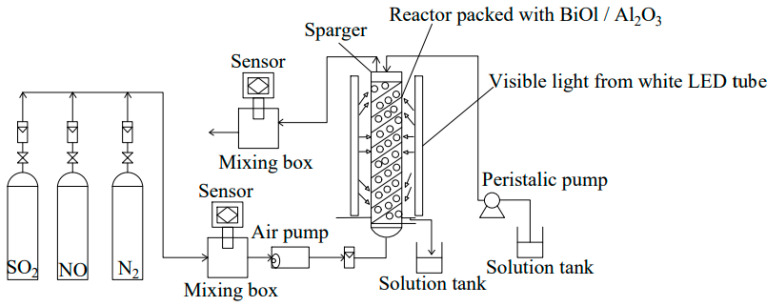
Process flow chart of photocatalytic desulfurization and denitrification integration [143].

The photocatalytic oxidation approach shows that when a photocatalyst is exposed to light of a specific wavelength, it catalyzes the excitation of surrounding water and oxygen molecules, leading to the formation of reactive ·OH and ·O_2_ free ion groups (Figure 16). These free ions oxidize gaseous pollutants, which can then be absorbed by a wet scrubber. This process not only oxidizes SO_2_ to sulfate but also converts NO and NO_2_ to nitrate, thereby accomplishing both desulfurization and denitrification [140]. Photocatalytic oxidation offers benefits such as a compact design, absence of secondary pollution, and high removal efficiency. TiO_2_, as a photocatalyst, absorbs light energy and can adsorb SO_2_ through van der Waals forces present between SO_2_ and TiO_2_ molecules. ZnO shares similar advantages with TiO_2_, including strong photo-oxidizing capabilities, good stability across a broad pH range, and an environmentally friendly profile. Man et al. prepared p-n junction ZnO-CuO/rGO ternary catalysts via the co-precipitation route [141]. The photocatalytic performance of ZnO-CuO/rGO was assessed for concurrent desulfurization and denitrification processes, achieving a desulfurization efficiency of 97% and a denitrification efficiency of 64% over a period of 270 min. When added to a seawater scrubber, ZnO-CuO/rGO facilitated a SO_3_^2−^ conversion rate of 80.72%. The photocatalytic technology is still mainly in the laboratory research stage, but the photocatalytic technology is a very desirable and promising technology for the treatment of ship exhaust. The process flow of photocatalytic desulfurization and denitrification is shown in Figure 17.

Currently, the widely respected process in the field of ship exhaust treatment is mainly the integrated technology that integrates the functions of desulfurization and denitrification. The main comparison among the most commonly used desulfurization and denitrification systems is given in Table 2. Compared to separate desulfurization and denitrification processes, integrated desulfurization and denitrification reduce the amount of space taken up by the ship through integrated design and improve space utilization, as well as being able to treat both SO_X_ and NO_X_ at the same time, thereby improving energy efficiency and increasing fuel utilization of the ship.

## 5. Conclusions and Recommendations

As the global economy and tourism develop rapidly, attention has been paid to the air pollutant problem caused by ship exhaust emissions. Following the introduction of Annex VI of the MARPOL convention in 1997, increasingly strict emission regulations have been put in place to curb emissions from ships. Consequently, numerous emission reduction methods have been explored and developed under various conditions and different devices. This paper is intended to provide a review of the status of emissions and control regulations for ship exhausts in the Lijiang River, and strategies for reducing ship diesel engine emissions. They are summarized as follows:

(1) The Lijiang River has a rich tourist landscape endowed by nature. A large number of tourists have caused a large amount of polluted gas emissions while on the cruise on the Lijiang River. Most ships in the Lijiang River use traditional low-speed diesel engines, with a large number of old ships, and the gas treatment of ships started late, and the equipment was backward. In order to improve air quality, the Guilin Maritime Safety Administration has actively taken measures to solve the problem of ship exhaust pollution in the Lijiang River. In addition to formulating strict emission regulations, the adjustment of ship structure, the promotion of new energy and the construction of a monitoring system are all important measures;
(2) SO_2_ and PM emissions are largely dependent on the sulfur content of the fuels used by ships. NO_X_ is mainly related to the combustion of nitrogen in the air when the engine is operated at high temperatures. Ships have become a major pollution source in certain inland rivers where shipping lanes are densely populated and ship traffic is high. NO_X_, SO_X,_ and PM emissions from ships can negatively impact the natural environment and human health along the coastline;
(3) Installing an exhaust aftertreatment system can effectively reduce NO_X_ and SO_X_ emissions, thereby meeting the Tier III standards. The wet flue gas desulfurization system can effectively capture SO_2_ from flue gas, concentrating it in gypsum and solution. The wet flue gas desulfurization system is applied to large two-stroke marine diesel engines operated with high-sulfur fuel. SCR is the most mature and effective exhaust treatment method for controlling marine diesel engine NO_X_ emission. Most of the exhaust aftertreatment techniques are mature, but they need to be used with appropriate integration and combination to achieve co-reduction in all pollutants and cost-effectiveness. Integrated desulfurization and denitrification treatment technology has its own advantages and disadvantages. The market has not yet found the most ideal solution and still needs to select the appropriate program in accordance with the specific needs of the ship exhaust treatment.

To address the challenges of rising ship emissions and stricter emission standards, the Lijiang River region must urgently adopt advanced exhaust treatment technologies. Current methods are limited by high energy consumption, costs, and spatial requirements. Future developments should focus on creating efficient, compact, and cost-effective solutions. The region should prioritize low-sulfur fuels, LNG, hybrid and electric propulsion systems, and intelligent management to optimize navigation and reduce emissions. New regulations will drive the industry toward cleaner practices, ensuring environmental sustainability and fostering a greener maritime sector.

## Figures and Tables

**Table 1:** Contribution rate of pollutant emission from ships in some regions [45,46].

Vintages	Districts	NO_X_	SO_2_	PM	VOC_S_	CO	SO_2_	PM_10_	PM_2.5_
2019	Jiangsu	18%				2.1%		4%	3.5%
2018	Dalian	2.4%	7.5%						7.1%
2018	Shenzhen	63%	23%		3%	5%		3%	3%
2015	Zhuhai	20%	17%		3%				10%
2012	Hong Kong	32%			11%	17%	50%	37%	43%
2007	Hong Kong	17%					11%	16%	
2010	Shanghai	11.6%					12.4%		5.6%

**Table 2:** Process characteristics and removal efficiencies of flue gas desulfurization and denitrification technology.

Processing	Advantages	Disadvantages	Removal Efficiency	Ref.
Dry desulphurization	No water consumption, environmentally friendly	Takes up a lot of space and by-products are difficult to handle	SO_2_: 85%	[144]
Semi-dry desulphurization	Low water consumption, environmentally friendly, simple process, small footprint	Lower desulfurization efficiency, higher calcium-to-sulfur ratio, higher raw material costs	SO_2_: 85%	[145]
Open type Seawater method	Uses seawater, no chemicals added	For low-sulfur fuels only Emissions fail to meet new standards	SO_2_: 95%	[146]
Magnesium method	Low up-front investment, simple process, easy maintenance, no scaling of products [147]	The use of alkaline chemicals increases costs.	SO_2_: 95–98%	[148]
Closed type Sodium alkali method	Low fouling, high SO_2_ absorption rate, avoid blockage of absorption tower	Not suitable for large amounts of use, large investment, large area, high operating cost	SO_2_: 99%	[149]
limestone-gypsum method	Mature technology, not easy to clog	The device covers a large area, the equipment is easy to wear and block	SO_2_: >90%	[150]
Ammonia method	It has a certain economic value and does not emit carbon dioxide or cause secondary pollution	High cost, takes up a lot of space, ammonia is a hazardous material and easy to corrode pipelines.	SO_2_: 99%	[151]
SCR denitrification technology	Mature technology, very high denitrification efficiency, easy maintenance, no secondary pollution, simple equipment design	Highly affected by SO_2_, only suitable for low-sulfur fuels	NO_X_: 96.1%	[152]
SNCR denitrification technology	Easy to install, simplicity, the catalyst-free system, wide range of applications, lower cost	High emission temperatures, low reductant utilization, and high window temperatures	NO_X_: 88.2%	[153]
Ozone oxidation denitrification	Occupying less space, easy to obtain the raw materials for the reaction, no fouling of the reaction products, strong selectivity, and fast oxidation speed	High energy consumption, high consumption of O_2_, and high cost	NO_X_: 90.3%	[117]
Electrostatic spray technology	Strong ability to remove particles, strong ability to remove sulfur and denitrification, occupies little space, environmental protection, the raw materials are inexhaustible, saves costs	High energy consumption, requires electrolysis of seawater.	NO_X_: 52% SO_2_: 43%	[154]
Plasma oxidation process	High desulphurization and denitrification rates, produce economic products	The equipment is expensive and energy-consuming, radioactive, and the product takes up a lot of space, O_2_, CO_2_, and H_2_O have a negative impact on the effectiveness	NO_X_: 86.9% SO_2_: 100%	[155]
Oxidative absorption	Strong oxidation capacity, economic and environmental protection, simple process flow, high removal efficiency, and easy modification of wet flue gas desulfurization equipment	Chemically unstable and difficult to handle with high-temperature gases, competitive absorption between NO_X_ and SO_2_ with oxidants, over oxidation	NO_X_: 85.0% SO_2_: 97.6%	[156]
Integrated UV-enhanced active chlorine process	Desulphurization rate of 100%, low system complexity, high degree of automation, wide range of raw material sources, no need to store, save ship space, safety. The strong and non-selective oxidation characteristics	The denitrification rate is 50–61 percent, requiring secondary denitrification. large energy consumption, competition for pollutant removal	NO_X_: 89% SO_2_: 99%	[157]
Photocatalysis	No need to add additional chemicals, selective specificity for more precise and efficient treatment, a compact structure, with no secondary pollution, and a high removal efficiency.	Influenced by the light factor, the absence of light is the need for additional energy, high requirements for catalyst stability, and high reaction conditions.	NO_X_: 98.9% SO_2_: 87.1%	[122,158]

## Data Availability

Data will be made available on request.
